# The Extracellular Matrix Influences the miRNA Landscape of Human Mesenchymal Stromal/Stem Cells

**DOI:** 10.3390/ijms26188830

**Published:** 2025-09-10

**Authors:** Roman Ushakov, Elena Burova

**Affiliations:** Institute of Cytology, Russian Academy of Sciences, 194064 Saint Petersburg, Russia

**Keywords:** mesenchymal stromal/stem cells (MSCs), decellularized extracellular matrix (dECM), secretome, miRNA, miR-146a-5p, MSCs niche, preconditioning

## Abstract

Mesenchymal stromal/stem cells (MSCs) are known to secrete a wide range of pleiotropic molecules promoting tissue repair and regeneration. Recent advances in cell sheet technology have demonstrated significant improvements in the regenerative capacity of MSCs within the sheet, retaining appropriate microenvironmental cues, and have suggested an instructing role of extracellular matrix (ECM). We previously found that the secretome of MSCs cultured on a decellularized MSC-derived ECM (dECM) was significantly enriched in dozens of cytokines, chemokines and growth factors compared to the secretome of MSCs grown on standard plastic dishes. The enriched secretome has been shown to have enhanced chemotactic and angiogenic properties, stimulate C2C12 myoblast proliferation and promote skeletal muscle regeneration in a murine in vivo model. Here, we report novel findings about dECM-induced changes in the miRNA profile of MSCs. We performed miRNA-seq and found 17 differentially expressed miRNAs in endometrial MSCs (MESCs) with miR-146a-5p being the most upregulated. Additionally, we investigated miR-146a-5p expression in MSCs of various origins after exposure to dECM, and found a correlation between miR-146a-5p upregulation and the general dECM-induced paracrine response. Furthermore, we demonstrated that miR-146a-5p mimics, transfected into C2C12 myoblasts, promoted their proliferation, suggesting a role for miR-146a-5p in myotropic effects mediated by the enriched secretome. These findings provide new insights into how ECM as a component of the MSC niche influences the secretory phenotype and modulates therapeutic properties of MSCs.

## 1. Introduction

Mesenchymal stromal/stem cells (MSCs) have been intensively studied for decades as cell therapy tools due to their pleiotropic medicinal effects, variety of sources and ease of isolation, culture and handling [1]. The therapeutic capacity of MSCs is primarily determined by secretion of multiple bioactive molecules including growth factors, hormones, cytokines, nucleic acids and lipids that exert immunomodulatory, anti-inflammatory, anti-oxidant and angiogenic effects, contributing to tissue repair and regeneration [2].

The key challenge in MSC therapy is the fragility of such cells outside the native niche. The standard approach for MSC usage implemented in hundreds of studies involves the isolation of MSCs, in vitro expansion in order to produce the therapeutic dose, harvesting from the culture surface with proteolytic enzymes and the injection of cell suspension into the injury site. Such an approach is compromised due to anoikis—programmed cell death—following the cell’s detachment from the extracellular matrix (ECM) [3].

This constraint can be overcome with either using MSC-derived secretomes instead of cells or applying MSCs within ECM-based scaffolds, providing support and an appropriate microenvironment. An intriguing example of such a scaffold for MSCs is the cell sheet. The cell sheet is produced due to the MSCs’ self-organization during long-term culture and it consists of single or multiple MSC layers which are abundant in ECM proteins. MSC sheets were demonstrated not only to enhance the viability of MSCs but also to surpass the therapeutic effects of their secretomes [4,5,6,7]. For instance, an adipose tissue-derived MSC sheet compared to treatment with an MSC suspension or secretome was shown to induce scarless regeneration of cutaneous pressure ulcers including restoration of skin appendages [5].

These advances in the cell sheet technique allowed us to hypothesize that signals from ECM not only provide structural support for MSCs but also enhance their secretory activity. We found previously that the culturing of MSCs on cell-derived decellularized ECM (dECM) led to significant upregulation of various cytokines, chemokines and growth factors such as FGF-2, G-CSF, GM-CSF, HGF, IL-6, IL-8, MCP-1, CXCL-5, CXCL-6, GRO-α and GRO-β [8]. We also demonstrated that the enriched secretome had enhanced angiogenic and chemotactic properties, stimulated in vitro myoblast proliferation and promoted the repair of injured murine skeletal muscle [8,9].

The purpose of the current study was to clarify whether ECM influences the expression of miRNAs, another therapeutically significant class of molecules secreted by MSCs. miRNAs are small (18–22 nucleotides) non-coding RNAs that regulate gene expression, binding to promoters or mRNAs. Since miRNAs are generally considered to play a crucial role in multiple biological processes including development and tissue repair [10,11], we attempted to deepen the description of the ECM-induced secretome enrichment phenomenon with respect to miRNAs.

## 2. Results

### 2.1. Differential Expression of miRNAs in MESCs Cultured on dECM

**Figure 1:**
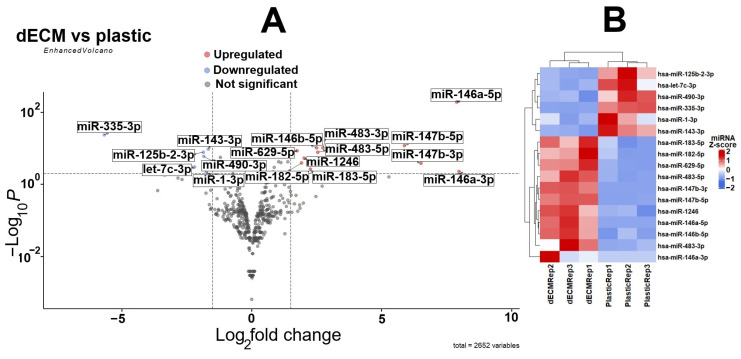
Results of miRNA-seq performed on endometrium-derived mesenchymal stromal/stem cells (MESCs) of three donors (*n* = 3) cultured on standard plastic and decellularized extracellular matrix (dECM) for 72 h. (**A**) Volcano plot represents differential expression of miRNAs (cultured on dECM versus cultured on plastic) according to fold change (FC) and padj (*p*-value adjusted for multiple comparisons). miRNAs were considered to be differentially expressed (indicated with red dots) if they matched two criteria (marked with vertical and horizontal dotted lines): |Log_2_FC| > 1.5 and padj < 0.01. The complete table with Log_2_FC and padj values for all identified miRNAs is available in the Supplementary Files (Table S1); (**B**) Heatmap depicts Z-score characterizing variability between donors.

We performed miRNA-seq analysis of endometrium-derived mesenchymal stromal/stem cells (MESCs) cultured on standard plastic and dECM. MESCs were chosen for miRNA-seq as they demonstrated the strongest upregulation of cytokines, chemokines and growth factors at both mRNA and protein levels upon maintenance on dECM [8]. Bioinformatic analysis revealed 17 differentially expressed miRNAs (|Log_2_FC| > 1.5 and padj < 0.01 thresholds were chosen as significant): 11 were upregulated and 6 were downregulated (Figure 1).

**Figure 2:**
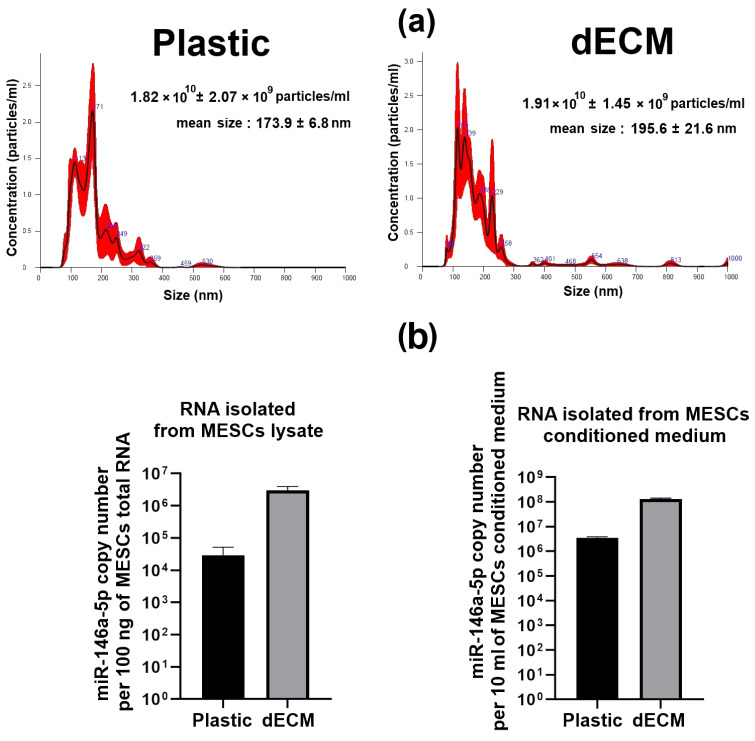
(**a**) Size distribution of nanoparticles detected with NanoSight NS300 analyzer in secretomes of MESCs cultured on dECM and plastic. Particle distributions are characteristic of exosomes. Data are presented as mean ± SD of three repeats (*n* = 3). (**b**) miR-146a-5p quantification using RT-qPCR in MESC lysates and conditioned media. miR-16-5p was used as the reference miRNA since its expression was stable according to miRNA-seq. Data are presented as mean ± SD of three repeats (*n* = 3).

According to miRNA-seq data, the most downregulated miRNA was miR-335-3p (about 50-fold), while miR-146a-5p and miR-146a-3p (about 235-fold) were the most upregulated. Next, we validated miR-146a-5p data using an RT-qPCR assay: We found that miR-146a-5p copy numbers were about 120-fold higher in lysates of MESCs cultured on dECM compared to ones cultured on plastic. In conditioned medium collected from MESCs cultured on dECM (CM-dECM), the copy numbers were about 40-fold higher than those in the secretome of MESCs cultured on plastic (Figure 2b). Thus, we confirmed that the secretome of MESCs cultured on dECM was significantly enriched in miR-146a-5p. We also hypothesized that culturing on dECM may intensify the production of exosome by MSCs in a similar way to cues boosting the secretion of vesicles, such as substrate stiffness and culture geometry [12,13]. However, we did not detect differences in exosome concentration between the secretomes of MESCs cultured on plastic and dECM (Figure 2a).

### 2.2. Differential miR-146a-5p Expression in MSCs Derived from Different Sources

**Figure 3:**
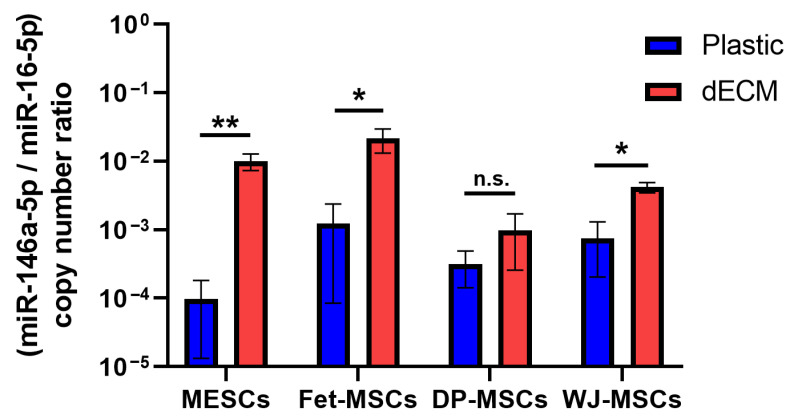
Differential expression of miR-146a-5p in MSCs derived from different sources cultured on dECM in comparison to ones cultured on plastic. Data are presented as means ± SD (*n* = 3) of miR-146a-5p copy number normalized to miR-16-5p used as the reference miRNA. *—*p* < 0.05; **—*p* < 0.01; n.s.—not significant (Student’s *t*-test). MESCs—MSCs derived from desquamated endometrium of menstrual blood; Fet-MSCs—MSCs of fetal bone marrow; DP-MSCs—MSCs derived from dental pulp; WJ-MSCs—MSCs derived from Wharton’s jelly of umbilical cord.

Next, we checked whether MSCs derived from various tissues would demonstrate dECM-induced miR-146a-5p upregulation. We found miR-146a-5p to be upregulated in MESCs, MSCs of fetal bone marrow (Fet-MSCs), MSCs of dental pulp (DP-MSCs) and Wharton’s jelly-derived MSCs (WJ-MSCs) cultured on dECM compared to the ones cultured on plastic. However, the upregulation degree was tissue-specific in MSC lysates (Figure 3): MESCs and Fet-MSCs exhibited a higher increase in miR-146a-5p expression (about 120-and 36-fold) while DP-MSCs and WJ-MSCs tended result in a lower increase (about 3- and 6.5-fold). Interestingly, these observations correlate with our previously published data about dECM-induced changes in the expression of cytokines, chemokines and growth factors in the same MSC types [8].

### 2.3. miR-146a-5p Mimic Promotes Proliferation of C2C12 Myoblasts

**Figure 4:**
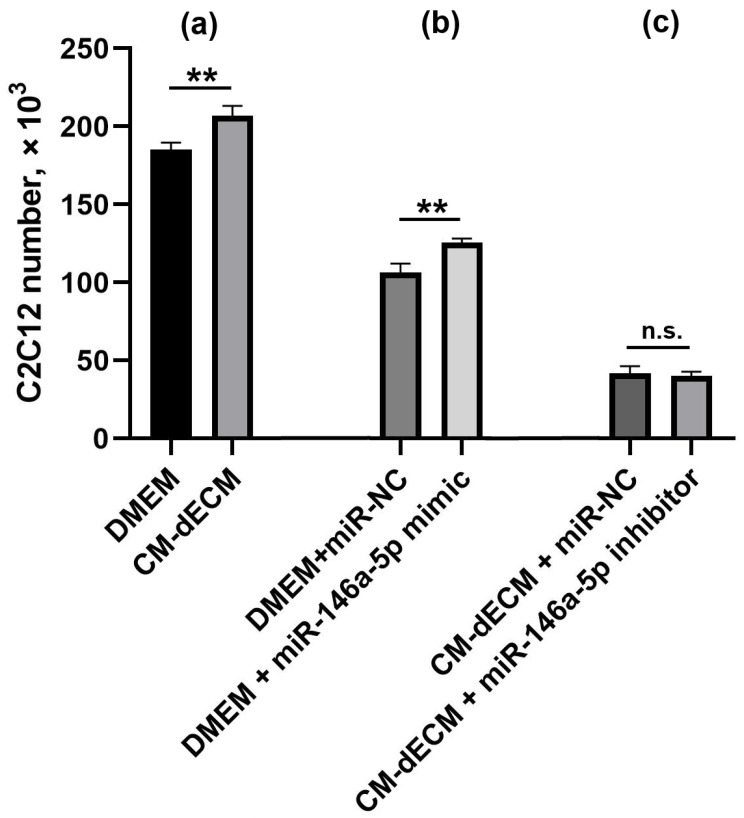
C2C12 cell number in groups cultured in: (**a**) DMEM and CM-dECM; (**b**) DMEM and transfected with a miR-146a-5p mimic or control microRNA; (**c**) CM-dECM and transfected with a miR-146a-5p inhibitor or control microRNA. Cell number was estimated 24 h after treatment. Values are presented as means ± SD (*n* = 3). **—*p* < 0.01; n.s.—not significant (Student’s *t*-test). CM-dECM—conditioned medium of MESCs cultured on dECM; miR-NC—negative control microRNA having minimal identity with miRNAs in humans and mice.

As we reported recently, enriched conditioned medium of MESCs cultured on dECM (CM-dECM) significantly enhanced the proliferation of C2C12 myoblasts and promoted skeletal muscle repair in a murine barium chloride injury model (Figure 4a) [9]. Although the secretome was shown to be enriched in several myokines such as FGF-2 and HGF known as regulators of myoblast proliferation [14], we hypothesized that miR-146a-5p, the most upregulated miRNA in MESCs’ secretome, may also participate in C2C12 proliferation. In order to test this hypothesis, we transfected C2C12 with a mature miR-146a-5p mimic. We found that 24 h after transfection, the cell number was significantly higher in the mimic-transfected group in comparison to the control miRNA-transfected one (Figure 4b). Additionally, we transfected C2C12 cultured in CM-dECM with a miR-146a-5p inhibitor in an attempt to attenuate C2C12 proliferation and validate the stimulating role of miR-146a-5p. Surprisingly, such transfection led to an idiosyncratic increase in cytotoxicity (Figure S2), and we did not observe the expected effect (Figure 4c).

## 3. Discussion

In the present work, we showed that MESCs cultured on dECM compared to those cultured on standard plastic lead to a significant shift in the expression profile of miRNAs, including 17 differentially expressed miRNAs. The concentration of the most upregulated one, miR-146a-5p, was shown to be increased 40-fold in the MESC secretome. miR-146a-5p is known as a potent regulator exerting anti-inflammatory and anti-fibrotic effects in various disease models via targeting the JAK/STAT, NF-κB and TGF-β/Smad pathways [15]. Moreover, MSC-derived exosomes loaded with miR-146a-5p mimic were demonstrated to ameliorate diabetic wound healing in mice through enhanced re-epithelization, angiogenesis and M2 macrophage polarization [16]. From this perspective, the dECM-induced boost of miR-146a-5p secretion appears to be a promising preconditioning approach to improving the therapeutic properties of MSCs.

As we reported previously, the MESC secretome enriched with multiple paracrine factors due to the culture on dECM remarkably stimulated the proliferation of C2C12 myoblasts and promoted skeletal muscle repair in a murine model of *m. tibialis anterior* necrosis [9]. Here, we show that transfection with the miR-146a-5p mimic facilitated C2C12 proliferation, suggesting a role for miR-146a-5p in the myotropic effects of the enriched MESC secretome. Interestingly, our results are in concordance with the report of Kuang et al., who found miR-146a to be upregulated in C2C12 subjected to cyclic stretch. Such an upregulation coincided with the inhibition of C2C12 differentiation into myotubes and with an increase in cell number, while transfection with the miR-146a inhibitor reduced these effects [17]. Intriguingly, Qin et al. investigated interactions between 3T3-L1 adipocytes and C2C12 myoblasts and reported the opposite effect: miR-146a-5p inhibited C2C12 proliferation and promoted its differentiation into myotubes [18]. The possible explanations remain to be speculated; nevertheless, all these findings provide evidence that miR-146a-5p exerts some effects on myoblasts.

We checked whether a dECM-induced shift in miR-146a-5p expression takes place in MSCs derived from different tissues. According to our data, MESCs and Fet-MSCs demonstrated more significant miR-146a-5p upregulation than WJ-MSCs and DP-MSCs. We reported previously that the same MSC types demonstrated similar dECM-induced enhancement of cytokines, chemokines and growth factor secretion [8]. Taken together, these findings allow us to hypothesize that MSCs’ receptivity to signals from dECM is tissue-specific, and variations in receptivity may be determined by differences in the expression profiles of receptors for ECM components. Considering the above-mentioned anti-fibrotic properties of miR-146a-5p, it could be proposed that MESCs manifest the most remarkable miR-146a-5p upregulation because endometrial cells are physiologically tuned to regenerate the functional layer of the endometrium without fibrosis on a monthly basis in order to provide an optimal milieu for embryo implantation. This hypothesis may be supported by the fact that menstrual blood serum contains some factors inhibiting endometrial fibrosis in vitro [19]; however, corroboration of this hypothesis requires further investigations of the presence and functionality of miR-146a-5p in endometrial fluid or menstrual blood.

Discussing hypothetical mechanisms underlying dECM-induced miR-146a-5p upregulation in MESCs, we found that 8 h after seeding, MESCs cultured on dECM accumulated significantly more phosphorylated NF-κB p65 (Ser536) in comparison to MESCs cultured on plastic; by 24 h, phospho-NF-κB p65 levels in MESCs cultured on plastic and dECM became equal (Figure S1). Considering that the promoter of miR-146a precursor contains two pairs of NF-κB binding motifs [15], miR-146a-5p in MESCs could be upregulated via NF-κB activation; however, the upstream signaling pathway and initial dECM-related stimuli remain unknown. Notably, miR-146a-5p can be regulated by mechanical stimuli: besides our findings regarding ECM-induced miR-146a-5p upregulation as well as results of Kuang et al., who showed an increase in miR-146a-5p expression after C2C12 cyclic stretch, miR-146a-5p was also reported to be upregulated after applying oscillatory pressure on small airway epithelium [20].

Several limitations of our study should be highlighted. The use of one time point (namely, 72 h after seeding the cells on dECM and plastic) as well as the sample size (*n* = 3) for miRNA-seq were limited due to high costs of the analysis. However, we showed previously that MESCs derived from the same donors demonstrated good consistency in dECM-induced upregulation of cytokines, chemokines and growth factors [8]. The next limitation is related to the nature of the dECM deposited by cultured cells. This dECM is a multicomponent mixture that complicates the search for stimuli that exactly influence the miRNA landscape of MSCs. At the same time, the strength of such an approach is that dECM mimics the interstitial matrix harboring MSCs in vivo. In addition, to avoid possible batch-to-batch variations in both the composition and properties of dECM, we tested every new dECM batch for cytokine and chemokine upregulation using RT-qPCR. The substantial limitation hindering potential therapeutic implications of our findings is that we focused on only the most upregulated miRNA, miR-146a-5p, while culturing on dECM led to differential expression of 16 more miRNAs. Many of them possess pleiotropic activities that should be kept in mind if the secretome or exosome fraction enriched via MSC culturing on dECM are intended for use as a therapeutic: for instance, miR-146a-3p and miR-146a-5p originating from the same precursor miRNA appear to exert opposite effects (pro-inflammatory and anti-inflammatory, respectively) [21]; miR-147b, the second most upregulated miRNA in MESCs, is characterized as oncomiR, promoting growth of several types of tumors [22,23]. However, without additional investigations it is hardly possible to assess the biological relevance of quantitative changes in each miRNA in the secretome of MSCs cultured on dECM. Finally, miR-146a-3p was found to be upregulated in MESCs on dECM, as it met the statistical criteria (Figure 1A). Based on the Z-score heatmap, which characterizes the variability in miRNA expression between donors, there was an outlier among the data points corresponding to miR-146a-3p expression in the dECM culture (Figure 1B). Therefore, miR-146a-3p may be mistaken for an upregulated miRNA and it requires further validation on a larger sample size.

In conclusion, we reported in the present work novel findings regarding ECM’s influence on the miRNA expression profile in MSCs. To our knowledge, there are no similar published data. Considering the importance of miRNAs as crucial molecules regulating development, tissue repair and regeneration on the one hand, and the significance of ECM for MSCs as reservoir of microenvironmental cues boosting their therapeutic capacity on the other hand, we believe that our work contributes to the field of MSC biology and provides a basis for further studies of ECM-MSC interactions. As a direction for future research, we suppose that the miRNA profiling of adipose tissue-derived MSC sheets could shed light on the mechanisms underlying the excellent improvement in its therapeutic properties in comparison to monolayer cultures or secretomes [5].

## 4. Materials and Methods

### 4.1. Cell Culture

Primary MESCs, Fet-MSCs, DP-MSCs, WJ-MSCs and C2C12 myoblasts were obtained from the shared research facility at the Vertebrate Cell Culture Collection of the Institute of Cytology, RAS. All MSCs were previously characterized according to the minimal criteria for defining MSCs proposed by the International Society for Cellular Therapy [24]; namely, all studied MSCs demonstrated multilineage differentiation potential, and they were shown to have positive CD73, CD90 and CD105 expression and a lack of CD34 and CD45 surface markers [25,26,27,28]. All cells listed above were cultured in DMEM/F12 growth medium (Gibco, Grand Island, NY, USA) supplemented with 10% fetal bovine serum (FBS, HyClone, Pasching, Austria), 1% penicillin-streptomycin (Biolot, Saint Petersburg, Russia) and 1% GlutaMax (Gibco, Grand Island, NY, USA) at +37 °C in a humidified incubator with 5% CO_2_. MSCs were subcultured 1:3 every 72 h with 0.05% EDTA-trypsin solution (Gibco, Grand Island, NY, USA) within 6–12 passages while C2C12 myoblasts were subcultured 1:5.

### 4.2. Preparation of dECM

dECM-coated culture dishes were prepared as described previously [8]. Briefly, 2 × 10^5^ WJ-MSCs were seeded into polystyrene Nunclon™ Delta 35 mm dishes (Thermo Fisher Scientific, Waltham, MA, USA) precoated with 0.1% bovine skin gelatin solution (Sigma-Aldrich, St. Louis, MO, USA) for 30 min at +37 °C. Then, the cells were cultured for 14 days in complete growth medium supplemented with 50 μg/mL 2-phospho-L-ascorbic acid trisodium salt (Sigma-Aldrich, St. Louis, MO, USA). Afterwards, the cells were lysed with 0.5% CHAPS (Sigma-Aldrich, St. Louis, MO, USA) with 20 mM ammonium hydroxide for 3 min at room temperature and washed three times with PBS. dECM-coated vessels were stored at +4 °C in PBS with 1% penicillin-streptomycin until further use.

### 4.3. Conditioned Medium Preparation

MESCs were seeded into control and dECM-coated dishes. Upon reaching 90% confluency, the cells were washed 5 times with PBS, and then 1 mL of serum-free DMEM/F12 medium supplemented with 1% penicillin-streptomycin and 1% GlutaMax was added. After 48 h, conditioned media were collected, centrifuged at 2500× *g* in order to eliminate cell debris, adjusted to have equal concentrations, sterilized with 0.22 μm PES syringe filters and frozen at −80 °C until further use.

### 4.4. miRNA-seq

MESCs obtained from three donors were cultured for 72 h on control and dECM-coated dishes. Next, total RNA was isolated with the use of phenol guanidine thiocyanate solution (ExtractRNA, Evrogen, Moscow, Russia) and purified on silica spin columns (Qiagen, Dusseldorf, Germany). Libraries for miRNA-seq were prepared by means of NEBNext^®^ Multiplex Small RNA Library Prep Kit for Illumina (New England Biolabs, Ipswich, MA, USA) according to the manufacturer’s instructions. Library quality control was performed with the Agilent Bioanalyzer 2100 (Agilent Technologies, Santa Clara, CA, USA) electrophoresis platform. Libraries were sequenced on an HiSeq 1500 (Illumina, San Diego, CA, USA). The bioinformatic pipeline was as follows: adapters were removed with cutadapt v. 3.1, reads were aligned to the reference genome (GRCh38) with bowtie v. 1.0.1 and reads associated with miRNAs were counted using featureCounts. Differential expression of miRNA was estimated with DESeq2 v. 1.42.1. Results were visualized with ggplot2 and EnhancedVolcano packages. Raw sequencing data and the raw count table are publicly available through the Gene Expression Omnibus (GEO) archives under accession number GSE301921.

### 4.5. miRNA RT-qPCR

Total RNA from MSCs and conditioned media for RT-qPCR was isolated in the same manner as described in the paragraph above. RT-qPCR was performed in a two-step assay by means of specific kits for qualitative assessment of miR-146a-5p and miR-16-5p (ALMIR, Algimed Techno, Minsk, Belarus) according to the manufacturer’s protocols. miR-16-5p was chosen as the reference miRNA since its expression did not change according to miRNA-seq-based differential expression analysis. Expression fold change values were calculated with use of Pfaffl’s equation [29]. miR-146a-5p copy numbers were estimated by means of synthetic calibrators supplied by the RT-qPCR kit manufacturer.

### 4.6. C2C12 Transfection with miRNA Oligonucleotides

1 × 10^5^ C2C12 myoblasts were seeded into 12-well plates in complete growth medium. After 24 h, upon reaching about 70% confluency, the cells were washed twice with PBS, the medium was changed to 500 μL serum-free DMEM or CM-dECM and the cells were transfected with 1 μL per well of GenJect-40 cationic lipid emulsion (Molecta, Moscow, Russia) and 100 mM miR-146a-5p inhibitor, 50 mM miR-146a-5p mimic and 50 mM miR negative control (Syntol, Moscow, Russia) according to the manufacturer’s recommendations. The negative control is derived from cel-mir-239b, and it has minimal identity with miRNAs in humans and mice. Oligos had full-length nucleotide 2′-methoxy modification. Oligo sequences were as follows: miR-146a-5p inhibitor 5′-AACCCAUGGAAUUCAGUUCU-3′, miR-146a-5p mimic 5′-UGAGAACUGAAUUCCAUGGGUU-3′, and miR negative control 5′-UUGUACUACACAAAAGUACUG-3′. At 24 h post-transfection, the cells were trypsinized and cell numbers were estimated by means of CytoFlex flow cytometer (Beckman Coulter, Fullerton, CA, USA).

### 4.7. Statistical Analysis

All experiments were performed at least in triplicate. Numeric data normality was checked with the Kolmogorov–Smirnov test. Groups were compared with Student’s *t*-test. *p*-values < 0.05 were considered as statistically significant. Statistical tests were carried out and visualized with GraphPad Prism v. 8.4.3 (GraphPad Software Inc., San Diego, CA, USA).

## Data Availability

The data that support the findings of this study are available from the corresponding author upon reasonable request. Raw sequencing data as well as the raw count table are publicly available through the Gene Expression Omnibus (GEO) archives under accession number GSE301921.

## Supplementary Materials

The following supporting information can be downloaded at https://www.mdpi.com/article/10.3390/ijms26188830/s1.

## Abbreviations

The following abbreviations are used in this manuscript:

CM-dECM	Conditioned medium derived from MESCs cultured on decellularized cell-derived extracellular matrix
dECM	decellularized cell-derived extracellular matrix
DMEM	Dulbecco’s modified Eagle medium
DP-MSCs	Mesenchymal stromal/stem cells isolated from dental pulp
ECM	Extracellular matrix
FC	Fold change
Fet-MSCs	Mesenchymal stromal/stem cells derived from fetal bone marrow
MSCs	Mesenchymal stromal/stem cells
MESCs	Mesenchymal stromal/stem cells derived from desquamated endometrium of menstrual blood
NC	Negative control
RT-qPCR	Reverse transcription–quantitative polymerase chain reaction
SD	Standard deviation
TEM	Transmission electron microscopy
WJ-MSCs	Mesenchymal stromal/stem cells isolated from Wharton’s jelly
