# Supplementary figures and images for: The Extracellular Matrix Influences the miRNA Landscape of Human Mesenchymal Stromal/Stem Cells
Source: Int J Mol Sci. 2025 Sep 10;26(18):8830. doi: 10.3390/ijms26188830 (PMC12470141; doi:10.3390/ijms26188830)

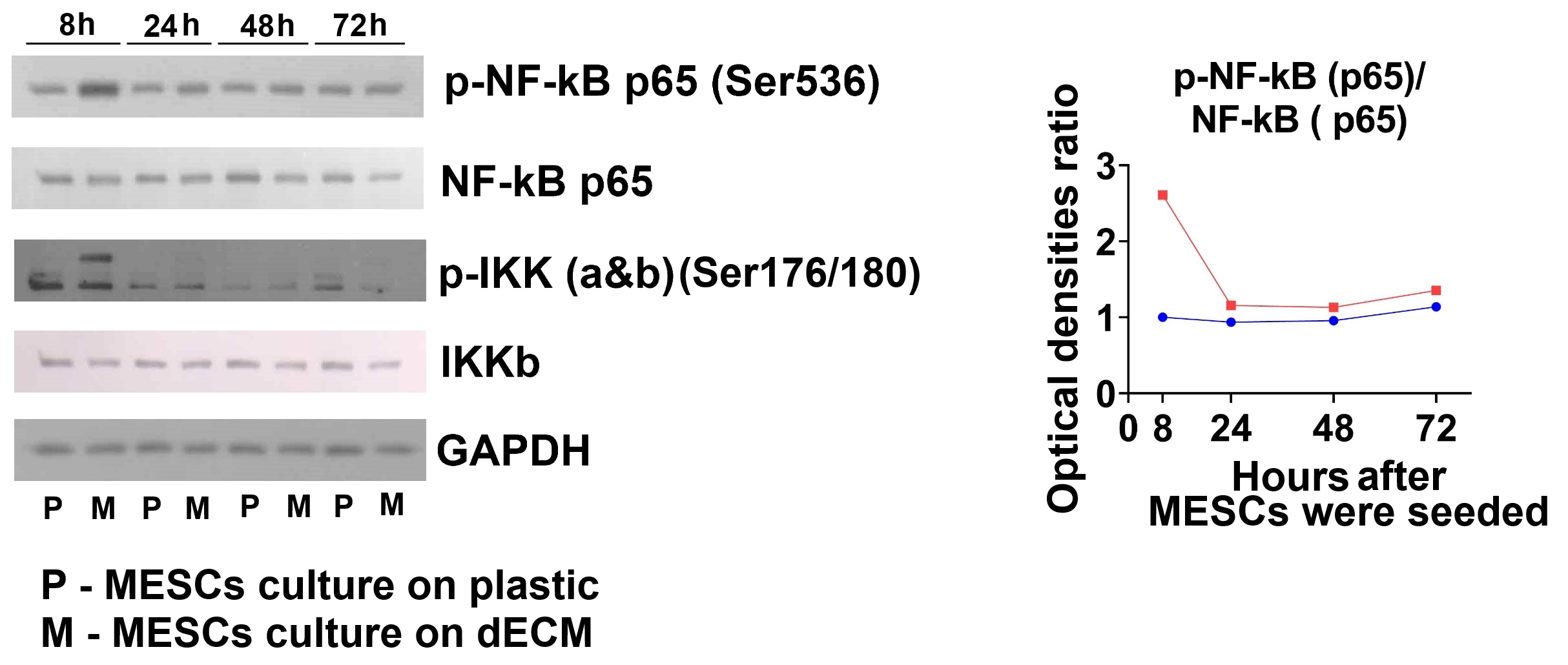

Supplement: Supplementary file 1 [file ijms-26-08830-s001.zip › Figure S1.jpg]

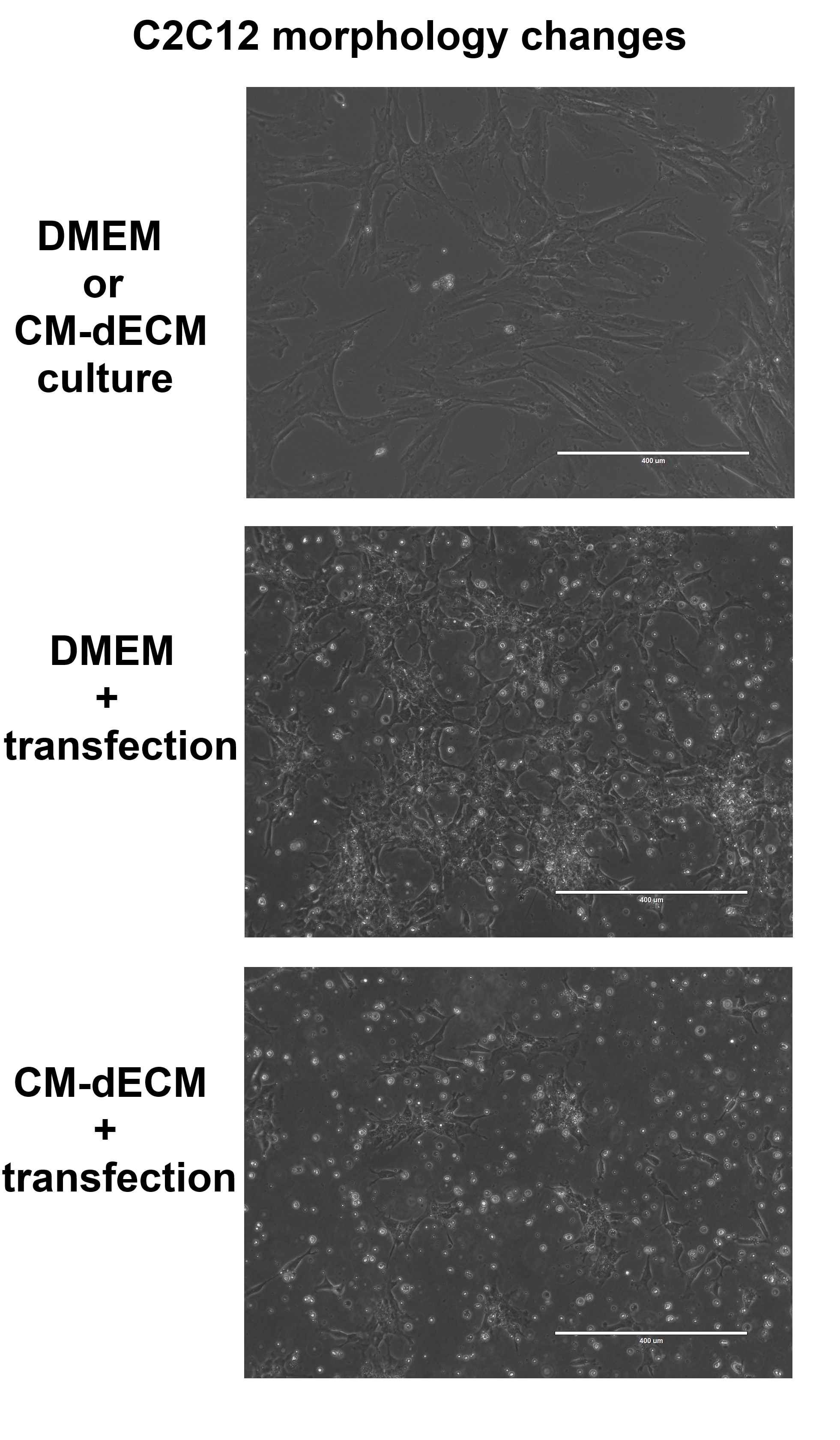

Supplement: Supplementary file 1 [file ijms-26-08830-s001.zip › Figure S2.tif]
